# Oral health in patients with cleft lip and palate: a systematic literature review and meta-analysis of periodontal and dental disease and oral microbiota (part 1)

**DOI:** 10.1186/s12903-025-05494-5

**Published:** 2025-01-29

**Authors:** A. Pardo, V. Vanti, F. Lonardi, A. Signoriello, G. Lobbia, G. Lombardo, L. Trevisiol, A. D’Agostino

**Affiliations:** 1 Dentistry and Maxillo-Facial Surgery Unit, Department of Surgery, Dentistry, Paediatrics and Gynaecology (DIPSCOMI), University of Verona, Piazzale L.A. Scuro 10, 37134 Verona, Italy; 2 Unit of Maxillo-Facial Surgery, Santa Chiara Regional Hospital, APSS, Trento, Italy; 3 Centre for Medical Sciences (CISMed), University of Trento, Trento, Italy

**Keywords:** Cleft lip and/or palate, Periodontal parameters, Oral microbiota

## Abstract

**Introduction:**

Orofacial cleft impacts jawbone and dental development and function, often with consequences for oral health. The first in this two-part systematic review of the literature on oral health in persons with cleft lip and/or palate focuses on periodontal parameters and composition of oral bacterial flora, while the second analyzes data on dental caries.

**Materials and Methods:**

Four databases (PubMed, Cochrane, Scopus, Web of Science) were searched for studies that compared periodontal parameters, caries index, and microbiota composition between persons with cleft lip and/or palate and healthy controls. The Newcastle–Ottawa scale and the Joanna Briggs Institute checklist were applied to evaluate study quality.

**Results:**

A total of 24 studies underwent systematic review, 18 of which entered meta-analysis. Twenty studies included a control group, while four included only persons with orofacial cleft. The first part (18 studies) compared periodontal parameters and oral microbiota.

**Conclusions:**

Meta-analysis of periodontal parameters (plaque index, gingival index, bleeding index, probing pocket depth, clinical attachment level) revealed an association between orofacial cleft and greater risk for poor oral health and periodontal disease. Good oral health relies on correct home oral hygiene and regular visits to the dentist starting at an early age.

## Background

### Rationale

Orofacial cleft, the most common congenital malformation in humans, results from disruption of growth and fusion of the frontonasal and the maxillary processes [1]. Its incidence is one in every 700 live births [2, 3]. Its etiology is unclear but is thought to be the final outcome of complex events involving environmental and genetic factors [3, 4]. Malformation can manifest with cleft lip and/or cleft palate and can be unilateral or bilateral; it can be isolated or part of a clinical syndrome (e.g., Pierre Robin syndrome, George syndrome, Treacher Collins syndrome) [2–4]. Orofacial cleft impacts the development and functionality of the jaw and the teeth, with serious implications for oral health. Optimal oral hygiene can be difficult to maintain due to anatomical defects associated with the cleft itself or alterations following surgery and orthodontic treatment, prosthetic implantation, postsurgical complications, e.g., oronasal fistula and/or residual scar tissue, and dental anomalies [4]. Such alterations of the oral cavity can promote colonization by microbial strains seldom found in healthy individuals and can disturb the homeostatic equilibrium between microorganisms and host [5]. Dysbiosis of the oral microbiota raises the risk for local infection besides the development of dental caries, periodontal disease [6], candidiasis, oral cancer [7], and airway disease, such as sinusitis and bronchiolitis [8].

### Scope and Objectives

For this two-part systematic review and meta-analysis of the literature we reviewed studies on oral health in persons with cleft lip or palate.

All types of cleft lip and palate were included in the review (cleft lip, cleft palate, cleft lip and palate with/without cleft alveolo, unilateral/bilateral clefts and other types). The research was not limited to a specific age group or the presence of any syndromes or surgical/orthodontic status. However, in cases where this information was not specified within the study, attempts were made to contact the authors to possibly carry out more specific analyses. Studies concerning oral health in patients with cleft lip and plate were selected, for which the following data were described:

- Quantitative data on caries experience in deciduous, mixed and permanent dentition (dmft, DMFT, dmfs, DMFS)
- Quantitative data on periodontal parameters (plaque indices, gingival inflammation indices, probing depth index, clinical attachment level index)
- Quantitative and/or qualitative data on the microbiota (bacteria, fungi).

The study objectives were to:

- Analyze the oral microflora of persons with cleft lip or palate and compare periodontal clinical parameters between persons with cleft lip or palate and healthy controls (Part 1).
- Compare differences in the prevalence of caries in deciduous, mixed, and permanent teeth between persons with cleft lip and palate and healthy controls as measured with epidemiological indices of caries; compare caries rating between different kinds of orofacial cleft (Part 2, upcoming manuscript).

## Methods

### Inclusion Criteria

Inclusion criteria were:

- full-text articles published in English involving human subjects, diagnosis of cleft lip or palate, description of oral health factors;
- quantitative data on dental caries in deciduous, mixed, and permanent teeth expressed as: DMFT (decayed, missing, filled teeth) and DMFS (decayed, missing, filled surfaces);
- quantitative data on periodontal parameters: plaque index, index of gum inflammation, index of depth of periodontal sounding, loss of clinical attachment level;
- quantitative or qualitative data on oral microbiota.

Exclusion criteria were:

- full text unavailable,
- publication in a language other than English,
- samples involving animals or in vitro,
- samples involving only subjects with apparatus for nasoalveolar molding,
- subjects undergoing presurgical orthopedic treatment, prosthesis wearers;
- case reports, letters, editorials, interviews, systemic reviews and meta-analysis of the literature.

### Information sources and search strategy

Four electronic databases were searched: PubMed, Scopus, Web of Science, Cochrane Library.

The search was conducted during the time interval from March 2023 to January 2024.

The following terms were used for the search strings: “cleft lip,” "cleft palate,” "oral microbiology,” "oral colonization,” "*Candida albicans*,” "DMFT,” "dental caries,” "plaque index,” "dental plaque,” "gingival index,” "bleeding on probing,” "gingivitis,” "periodontal disease,” "periodontal index.”. The Boolean operators “AND” and “OR” were used to connect the terms to limit the search and make it more efficient.

For all databases the following string was used:

- “Cleft lip" OR "Cleft palate"
- AND
- "Oral microbiology" OR "Oral colonization" OR "Candida Albicans" OR "DMFT" OR "dental caries" OR "plaque index" OR "Dental plaque" OR "gingival Index” OR "Bleeding on probing" OR "gingivitis" OR "periodontal disease" OR "periodontal index".

Studies that may have been initially missed were manually searched. For the present review, all types of labiopalatoschisis were included (labioschisis, palatoschisis, labiopalatoschisis with or without alveolar schisis, unilateral and bilateral schisis, and other types). The study was not limited to any specific age group, symptoms, or surgical/orthodontic state.

### Screening, selection and data collection

The screening and selection of studies were performed by two independent operators Two (AP, VV), and included: the removal of duplicates, the elimination of not accessible articles, manuscripts published in languages other than English. The titles and abstracts assessed as suitable from the records obtained were analyzed, excluding those not in line to the inclusion criteria. The full text of medical and/or orthodontic studies considered as relevant from the previous steps was read to assess their final inclusion in the review. In case of disagreement between the two reviewers, a third party (FL) intervened in the decision-making process.

Data collection from the included reports was performed by the same first two reviewers (AP, AS), who independently worked on an Excel spreadsheet to identify parameters referring to general article information (title, author, year of publication, journal, volume, and pages), study design, the population included, type of intervention, control used, and outcomes. No data extraction software was used.

### Data parameters

According to the objectives, primary outcomes assessed in the studies were:

- the following periodontal indices: Plaque Index (PI), Plaque Index Score (PIS), Visible Plaque Index (VPI), Quigley-Hein Index (QHI), Gingival Index (GI), Bleeding Index (BI), Bleeding on Probing (BoP) Probing Pocket Depth (PPD) and Clinical Attachment Level (CAL);
- the following caries indices: decayed missing filling teeth (dmft), decayed missing filled surfaces (dmfs), DECAYED MISSING FILLED TEETH (DMFT) and DECAYED MISSING FILLED SURFACES (DMFS).

Secondary outcomes concerned the analysis of the oral microbiota, to compare the main microorganisms between patients with schisis and healthy controls.

### Risk of bias assessment

Two reviewers independently applied the Newcastle-Ottawa scale (NOS) [9] and the Joanna Briggs Institute (JBI) [10] checklist to determine and classify study quality. Disagreement was resolved by discussion.

The Newcastle-Ottawa scale (NOS) [9] is a tool for assessing the quality of non-randomized studies. The evaluation includes questions on three domains: selection of study groups, comparability of groups, assessment of exposure or outcome of interest. Each question, based on the answers, can be assigned one star (with a maximum of 4 stars [****] for selection, with a maximum of two stars [**] for comparability, with a maximum of three stars [****] for the result). The quality of a study is calculated based on the sum of the stars for a maximum of 9 stars. Higher scores indicate lower risk of bias. Studies are considered of high quality when the overall score is >=7, they are considered of moderate quality when the score is between 4 and 6, and they are considered of low quality when the score is <=3.

The Joanna Briggs Institute (JBI) [10] is a tool used for cross-sectional, case-control and cohort studies, with a collection of different questions focused on the appropriateness of the statistical analysis used (Y yes, N no, UC unclear).

### Effect measures

Continuous outcomes were identified for the meta-analysis. The number of samples, mean, and standard deviation were defined for each outcome.

### Statistical analysis

Statistical analysis was conducted on studies with comparable outcomes, while microbiological analysis was not included in the meta-analysis because data comparison would have been practically impossible. Statistical analysis of periodontal parameters and caries indices was performed.

Review Manager Web software (Cochrane Collaboration) was used for analyzing the quantitative data. Meta-analysis was performed using a fixed-effects model, inverse-variance method, average differences, or standardized average differences according to the studies and parameters. A 95% confidence interval was calculated for the primary results.

Finally, forest plots were created and heterogeneity and overall effect tests were performed. Subgroup analysis explored the causes of statistical heterogeneity and to differentiate studies involving different age groups. Method to quantify statistical heterogeneity was I^2^.

### Certainty assessment

The assessment of confidence was conducted using GRADEpro Guideline Development Tool (GDT). The two reviewers evaluated the level of confidence in the evidence based on the following factors: risk of bias, inconsistency, indirectness, imprecision, publication bias, large effect, plausible confounding, and dose-response gradient. In the event of a disagreement between the two reviewers, additional discussion was used to reach a consensus.

## Results

### Study selection

The search was conducted during the time interval from March 2023 to January 2024. The search retrieved 1350 records:

- 308 from PubMed,
- 581 from Scopus,
- 58 from Cochrane
- 403 from Web of Science.

A manual search yielded four additional studies.

A total of 163 duplicates and 12 studies published in languages other than English were removed. 918 studies were excluded using filters: 753 before 2013 (as deemed not complete for the variables considered and to provide an updated overview of the oral health of these patients), 5 not full text, 29 not conducted on humans, 114 reviews or meta-analyses and 17 chapters, conference papers and notes. 273 studies were assessed for eligibility, and 204 of these were excluded after reading the title/abstract. Of the 69 articles to be analyzed, 4 were excluded because they were not reachable. 65 studies were examined. After text analysis, 14 irrelevant articles and 15 articles that did not meet the inclusion criteria were excluded.

Finally, a total of 24 studies underwent qualitative review and subsequent metanalysis: these were full-text published in English after 1 January 2013, deemed as complete for the variables considered according to the inclusion criteria (involving human subjects, diagnosis of cleft lip or palate, description of oral health factors).

**Fig. 1:**
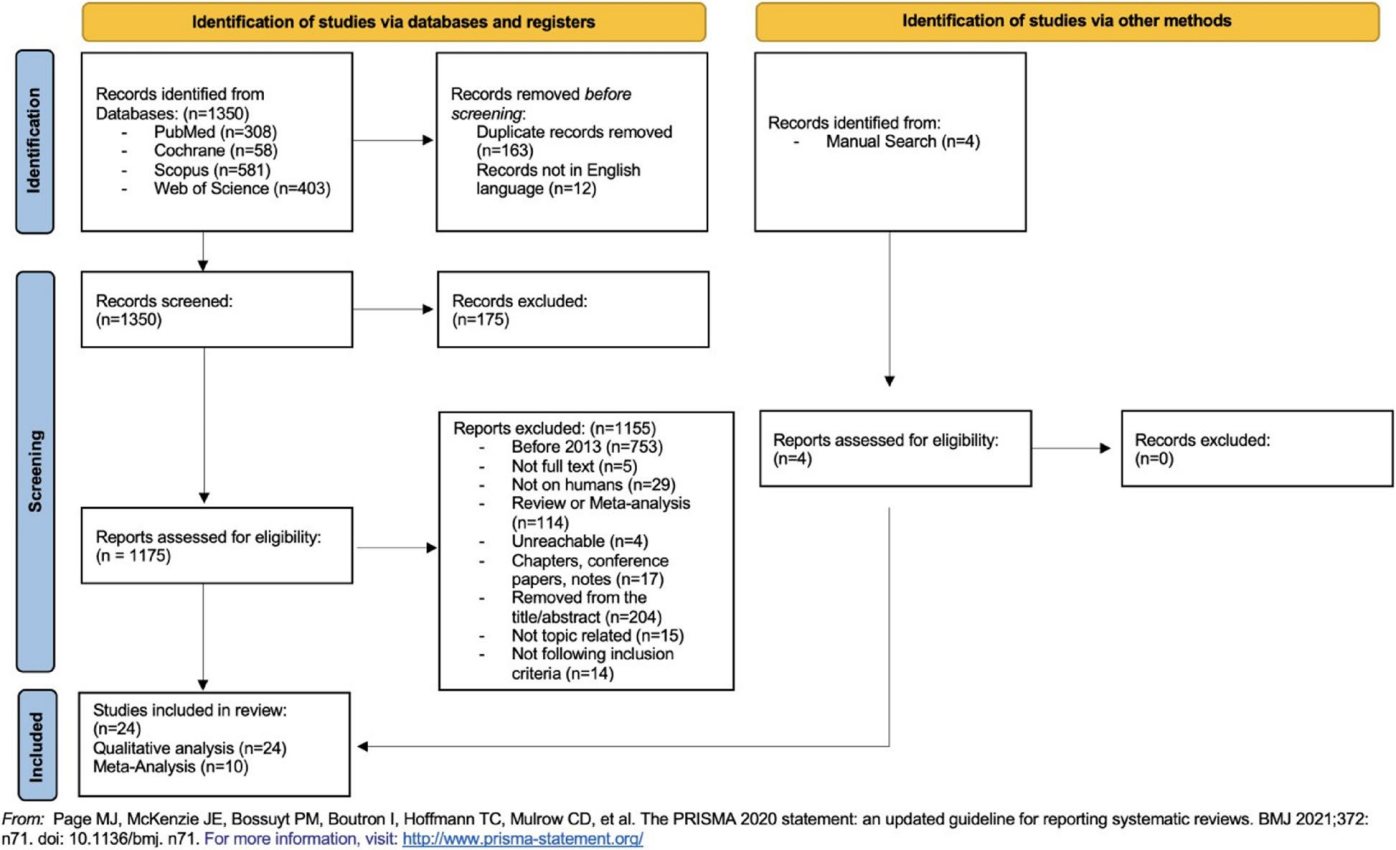
Study Flow Chart according to PRISMA criteria: search strategy and studies retrieved

Figure 1 presents the study flow chart according to PRISMA criteria, with number of articles retrieved and included.

### Study characteristics

Ten of the 24 studies (five case-control [11–15], four cross-sectional [16–19], one cohort [20]) evaluated clinical periodontal measurements in 1619 subjects (643 with lip cleft palate and 976 healthy controls) aged >2 years and three were conducted in Brazil [11, 14, 16], two in India [13, 15], two in Sweden [18, 19], one study each in China [12] and in Thailand [17] (Table 1). All defined the type of cleft.

**Table 1:** Studies characteristics: main findings

Author	Publication year	Country	Study Design	Cleft (N)	No-cleft (N)	Subject Age (yrs unless otherwise stated)	Cleft	Surgery	Orthodontics	Syndrome	Outcomes
King	2013	China	CC	132	132	2–4 & 5–7	CLP	NR	No	No	dmft
Freitas	2013	Brazil	CS	30	30	12–21	CL ± A, CP u/b CLP	Yes	Yes (18 with and 18 without cleft)	No	DMFT, BI, VPI
Kirchberg	2014	Germany	CS	295	548	1–6	CL, CP,CLP	NR	NR	No	dmft
Chopra	2014	India	CC	74	8	4–6	CLP	NR	NR	NR	dmft, GI, PI-S
Pisek	2014	Thailandi	CS	68	118	10–14	CL, CP, u/b CLP	NR	NR	No	PI,GI,dmft, DMFT
Sundell, Nilsson	2015	Sweden	CC	139	313	5–10	CL/P	NR	NR	Yes	dmfs, DMFS
Tuano	2015	Philippines	CS	332		2–5 & 6–9 & 10–12	CL, CP, CLP	NR	NR	No	dmft,DMFT
Sundell	2015	Sweden	CS	133	297	5 & 10	CL/P	NR	NR	Yes	dmfs (high/low risk),QHI, microbial analysis
Shashni	2015	India	CC	23	50	4–9	CLP	NR	No	No	Microbial analysis
Xiao	2015	China	CS	268		9–18	CL,CLP, CP	Yes	No	No	dmft, DMFT
Veiga	2016	Brazil	CC	78	78	5–18	CLP	Yes	Yes (35 with and 18 without cleft)	No	dmfs, DMFT,PI,BI, PPD, CAL
Kulas	2016	Germany	CC	73	73	8	CLP	Yes	NR	NR	dmft/DMFT
Kamble	2016	India	CS	500	500	5–15	u/b CL, CP, u/b CLP, CA	Yes/No	NR	NR	dmft, dmfs, DMFT, DMFS
Sunderji	2017	USA	CC	61	122	2–6	u/b CLP	NR	NR	No	dmfs, PI
Liu	2017	China	C	35	41	12–19	CL,CP	Yes	NR	NR	PI, GI, PPD, CAL
Sundell	2018	Sweden	CS	80	144	5	CL ± P	NR	NR	Yes	dmfs, microbial analysis, QHI, BOP
Durhan	2018	Turkey	CS	21	13	0–3	CP ± L	NR	No	No	Microbial analysis
Nagappan	2019	India	CS	80	80	8–16		No	NR	NR	DMFT
Fowler	2020	New Zeland	CS	554		5 &12	CL, CLP, CP	NR	NR	NR	dmft, DMFT
Allam	2020	Egypt	CS	120		4–12	CL ± A, u/b CLP	Yes	NR	No	dmft,DMFT
Gheller	2021	Brazil	CC	60	58	6–18	CL/P	Yes	NR	No	PI,BI, PPD, CAL, microbial analysis
Zaira	2021	Russia	C	70	60	8 mths—3 yrs	CL, CP	Yes	No	NR	Microbial analysis
Boriollo	2022	Brazil	CS	44	52	CLP: Females 18 ± 14.5; Males 12.8 ± 9.2; CTRL: Females 25 ± 7.0; Males 23.9 ± 7.1	CBT,CP-FC, CP-FBI, CP-FC, CP-FLI, CP-FRI, CP-FLC, CP-FR, CTLC,CTRC	Yes/No	NR	NR	Microbial analysis
Khan	2023	India	CC	50	50	< = 5, 6–16, > = 17	u/b CLP, CP, CP (soft), CA	Yes	NR	No	dmft, DMFT, PI, GI, microbial analysis

*NR* not reported, *CC* Case–control study, *CS* Cross-sectional study, *C* Cohort study, *CL* Cleft lip, *CP* Cleft palate, *CLP* Cleft lip and palate, *u/b CLP* unilateral/bilateral cleft lip and palate, *CL* ± *A* cleft lip ± alveolus, *CP (soft)* Cleft palate soft, *CA* Cleft alveolus, *CBT* cleft bilateral transforaminal, *CP-FC* cleft post-foramen complete, *CP-FBI* cleft pre-foramen bilateral incomplete, *CP-FC* cleft post-foramen complete, *CP-FLI* cleft pre-foramen left, *CP-FRI* right incomplete, *CP-FLC* cleft pre-foramen left complete, *CP-FRI* cleft pre-foramen right complete, *CTLC* cleft transforamen left, *CTRC* cleft transforamen right, *dmft/DMFT* Decayed missing filled teeth, *dmfs/DMFS* Decayed missing filled surfaces, *GI* Gingival Index, *PI* Plaque Index, *PPD* Probing pocket depth, *CAL* Clinical attachment level, *VPI* Visible plaque Index, *BI* Bleeding Index, *PIS* Plaque index score, *QHI* Quigley Hein index

Four studies involved subjects who underwent surgery [11, 14, 16, 21], five did not state whether they did [12, 15, 17–19], one study reported that 16 out of 50 subjects with cleft lip and palate did not undergo surgery, 18 subjects underwent surgery twice, nine underwent surgery once, and seven underwent surgery three times [13]. Seven studies excluded patients with lip cleft and palate associated with history of a syndrome [12, 15, 17–19]. Two studies included subjects with syndromes [18, 19] and one did not specify [15]. Two studies included subjects (cases and controls) undergoing orthodontic treatment [11, 16], one study excluded subjects undergoing orthodontic treatment [14], and seven studies did not specify [12, 13, 15, 17–19, 21].

Eight observational studies analyzed oral microflora [13, 14, 18, 19, 22–25]. The studies included a mixed age group of patients with lip cleft and a healthy controls group. All reported on the type of cleft.

Four reported that subjects underwent surgery [13, 14, 22, 24], while four did not specify [19, 23, 25, 26]. Two studies included symptomatic subjects [22, 24], four excluded subjects with symptoms [13, 14, 23, 25], and two did not specify [22, 24]. Five studies did not specify whether the subjects were receiving orthodontic treatment [13, 14, 18, 19, 22] and three excluded subjects who were [23–25].

### Risk of bias

**Fig. 2:**
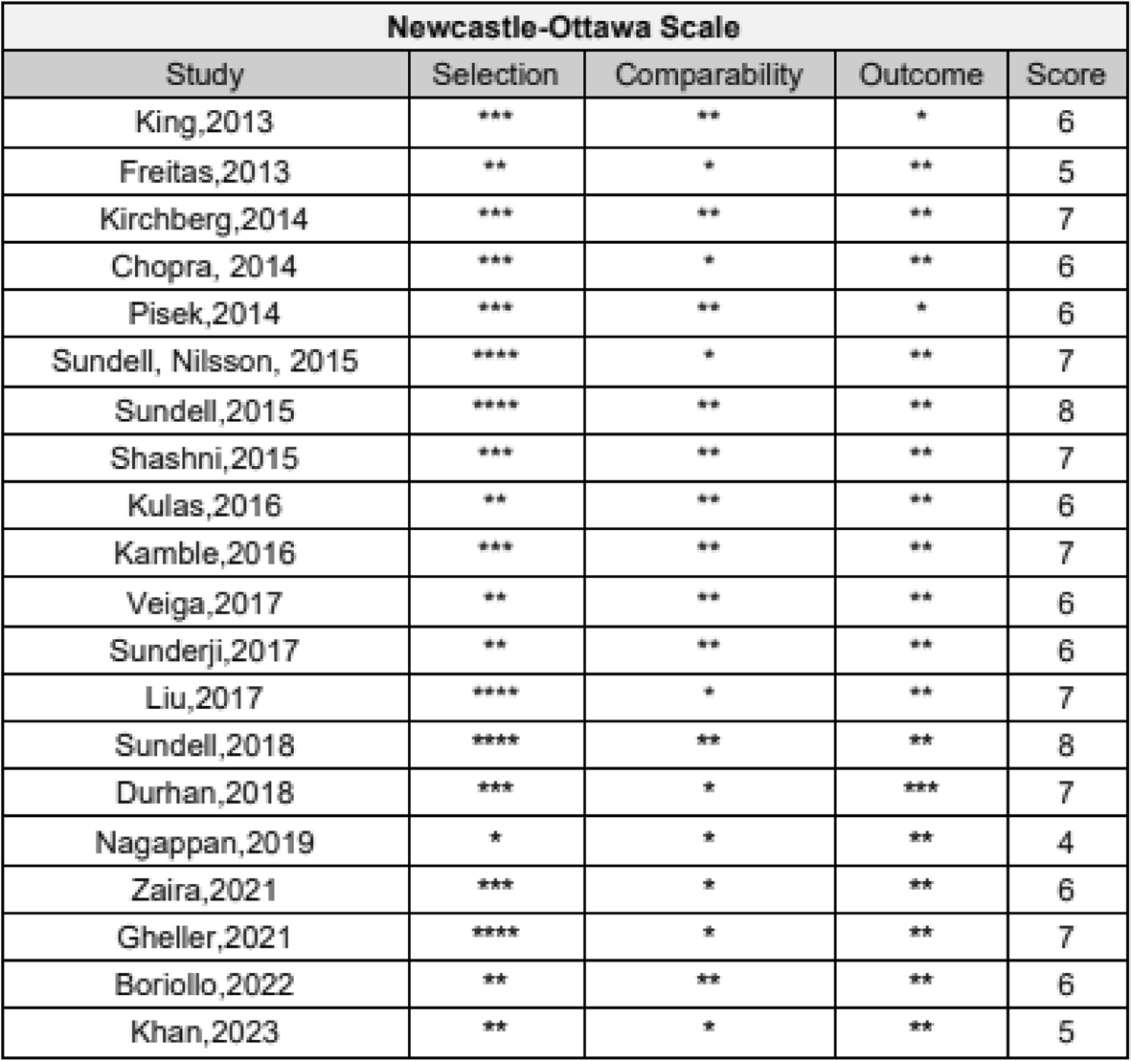
Newcastle–Ottawa scale (NOS) for assessing the quality of non-randomized studies. **** maximum score for selection and outcome, ** maximum score for comparability

**Fig. 3:**
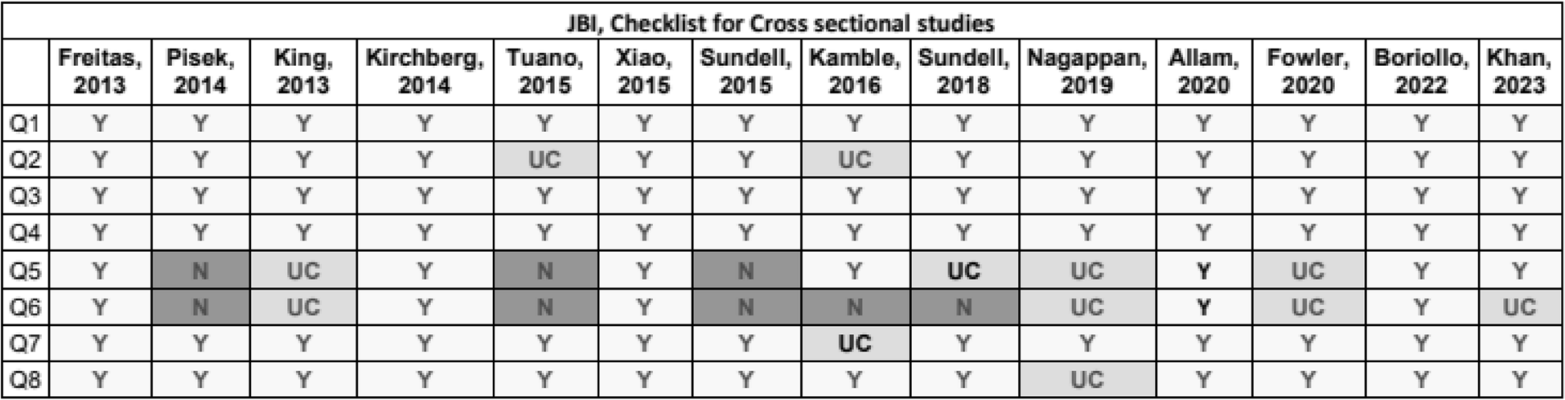
Joanna Briggs Institute checklist for assessing the quality of cross-sectional studies. Q1—Were the criteria for inclusion in the sample clearly defined?, Q2—Were the study subjects and the setting described in detail?, Q3—Was the exposure measured in a valid and reliable way?, Q4—Were objective, standard criteria used for measurement of the condition?, Q5—Were confounding factors identified?, Q6—Were strategies to deal with confounding factors stated?, Q7—Were the outcomes measured in a valid and reliable way?, Q8—Was appropriate statistical analysis used? Y yes, N no, UC unclear

**Fig. 4:**
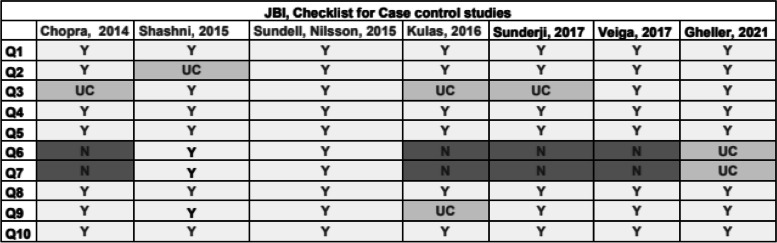
Joanna Briggs Institute checklist for assessing the quality of case–control studies. Q1—Were the groups comparable other than the presence of disease in cases or the absence of disease in controls? Q2—Were cases and controls matched appropriately?, Q3—Were the same criteria used for identification of cases and controls?, Q4—Was exposure measured in a standard, valid and reliable way?, Q5—Was exposure measured in the same way for cases and controls?, Q6—Were confounding factors identified?, Q7—Were strategies to deal with confounding factors stated?, Q8—Were outcomes assessed in a standard, valid and reliable way for cases and controls?, Q9—Was the exposure period of interest long enough to be meaningful?, Q10—Was appropriate statistical analysis used? Y yes, N no, UC unclear

**Fig. 5:**
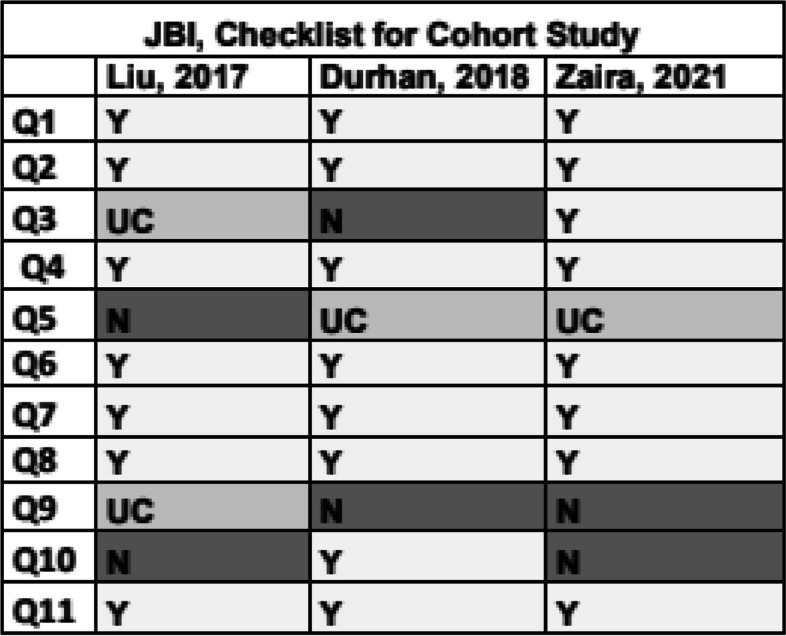
Joanna Briggs Institute checklist for assessing the quality of cohort studies. Q1—Were the two groups similar and recruited from the same population?, Q2—Was the exposure measured similarly to assign people to both exposed and unexposed groups?, Q3—Was the exposure measured in a valid and reliable way?, Q4—Were confounding factors identified?, Q5—Were strategies to deal with confounding factors stated?, Q6—Were the groups/participants free of the outcome at the start of the study (or at the moment of exposure)?, Q7—Were the outcomes measured in a valid and reliable way?, Q8—Was the follow-up time reported and sufficient to be long enough for outcomes to occur?, Q9—Was follow-up complete, and if not, were the reasons to loss to follow-up described and explored?, Q10—Were strategies to address incomplete follow-up utilized?, Q11—Was appropriate statistical analysis used? Y yes, N no, UC unclear

The studies were evaluated and classified for quality according to the Newcastle–Ottawa scale (NOS) [9] and the Joanna Briggs Institute (JBI) [10] checklist (Figs. 2, 3, 4 and 5).

### Meta-analysis

#### PI (Plaque Index) in patients with orofacial cleft and healthy controls

**Fig. 6:**
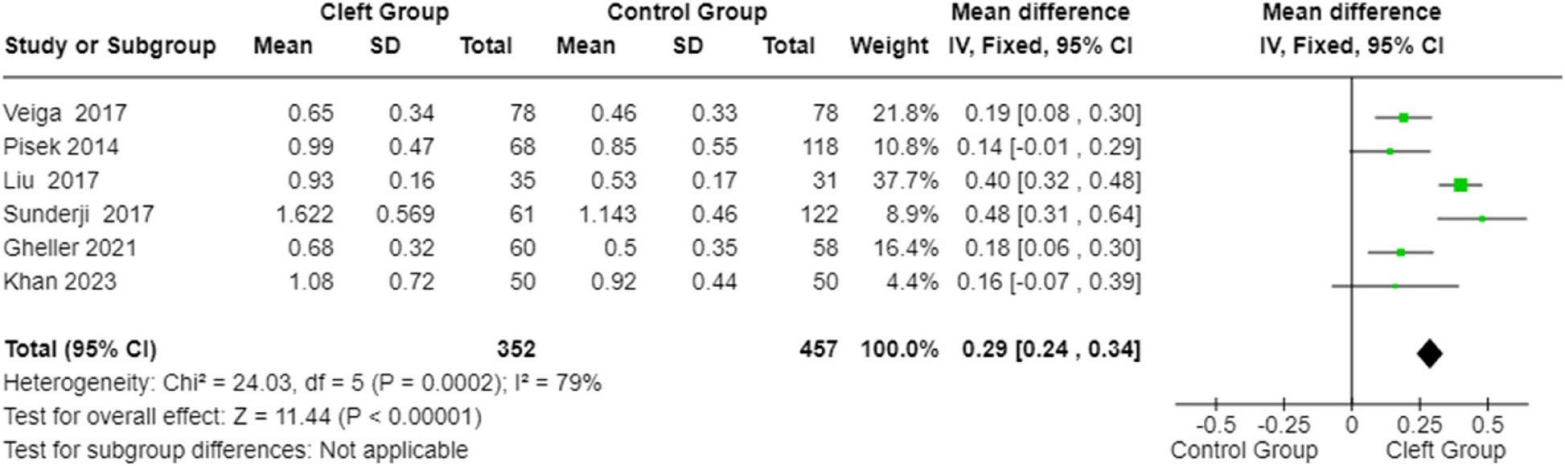
Forest plot for PI in patients with orofacial cleft and healthy controls

A statistical analysis was conducted on the PI parameter between subjects affected by cleft lip and palate and healthy subjects using the fixed effects model and mean differences. The 95% confidence interval calculated in the total observations is centered at 0.29 (with values ​​of 0.24–0.34). Both the heterogeneity test and the overall effect test are statistically significant (*p*-value:0.0002; *p*-value: < 0.00001). The forest plot (Fig. 6) showed a higher PI for the cleft group.

#### PI (Plaque index) in patients with unilateral and with bilateral cleft lip and palate

**Fig. 7:**
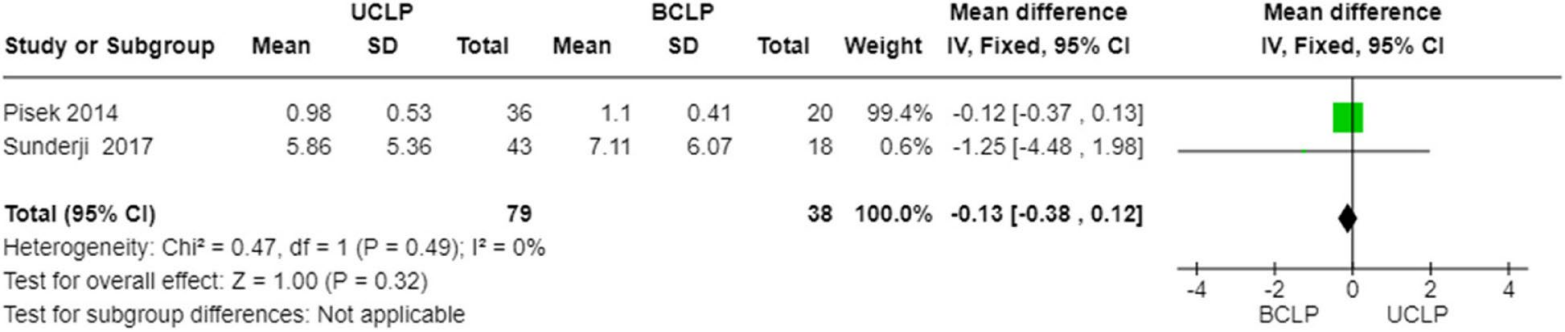
Forest plot for PI in patients with unilateral and with bilateral cleft lip and palate

A statistical analysis was conducted on the PI parameter between subjects affected by unilateral cleft lip and palate and subjects affected by bilateral cleft lip and palate using the fixed effects model and mean differences. The 95% confidence interval calculated across the total observations is centered at -0.13 (with values ​​of -0.38; 0.12). The heterogeneity test is not statistically significant (*p*-value:0.49). The overall effect test is not statistically significant (*p*-value:0.32). Comparison between unilateral and bilateral cleft lip and palate showed no statistically significant difference (Fig. 7).

#### GI (Gingival Index) in patients with orofacial cleft and healthy controls

A statistical analysis was conducted on the GI parameter between subjects affected by cleft lip and palate and healthy subjects using the fixed effects model and mean differences.

**Fig. 8:**
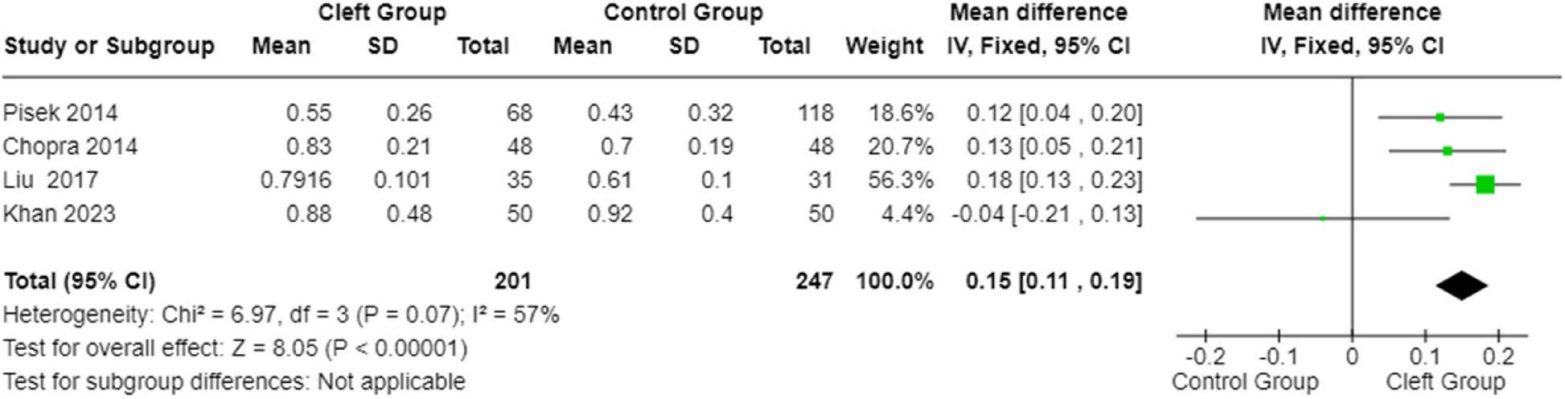
Forest plot for GI in patients with orofacial cleft and healthy controls

The 95% confidence interval calculated across the total observations is centered at 0.15 (with values ​​of 0.11-0.19). The heterogeneity test is not statistically significant (*p*-value:0.07), while the overall effect test is statistically significant (*p*-value:<0.0001). The gingival index (Fig. 8) was higher for the cleft group.

#### PPD (Probing pocket depth) in patients with orofacial cleft and healthy controls

**Fig. 9:**
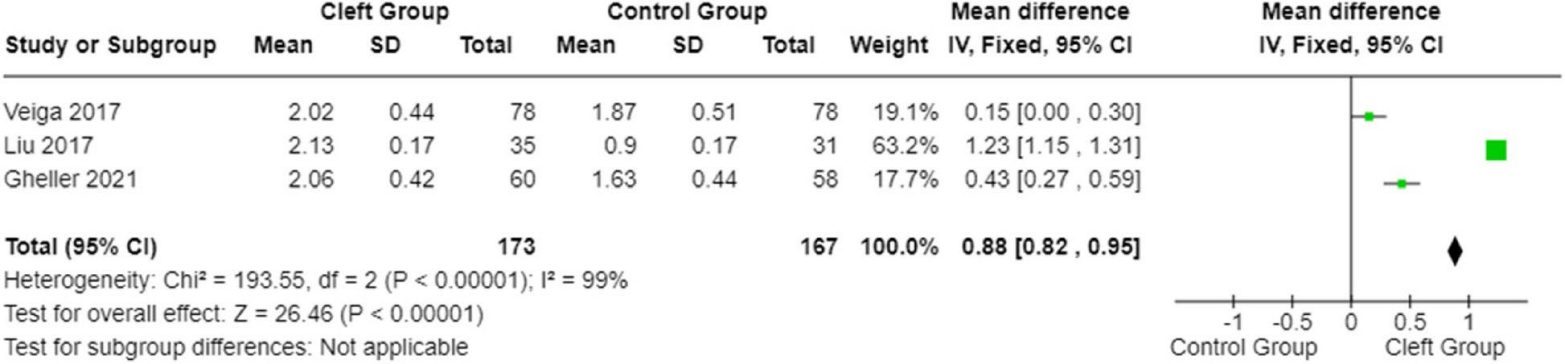
Forest plot for PPD in patients with orofacial cleft and healthy controls

A statistical analysis was conducted on the PPD parameter between subjects affected by cleft lip and palate and healthy subjects using the fixed effects model and mean differences. The 95% confidence interval on the total observations is centered at 0.88 (with values ​​of 0.82-0.95). Both the heterogeneity test and the overall effect test are statistically significant (*p*-value:<0.00001; *p*-value:<0.00001). The probing pocket depth (Fig. 9) was higher for the cleft group.

### Certainty assessment

The estimated risk of bias displayed Low certainty evidence, with comparisons between the two intervention tests and controls, for PI, GI and PPD (Table 2).

**Table 2:** Grade evidence table

Certainty assessment							№ of patients		Effect		Certainty	Importance
**№ of studies**	**Study design**	**Risk of bias**	**Inconsistency**	**Indirectness**	**Imprecision**	**Other considerations**	**cleft lip and/or palate**	**healthy subjects**	**Relative** **(95% CI)**	**Absolute** **(95% CI)**		
**Plaque Index**												
6	non-randomised studies	not serious	not serious	not serious^a^	not serious^b,c,d^	none	809	809	-	MD **0.29 SD higher** (0.24 higher to 0.34 higher)	⨁⨁◯◯ Low^a,b,c,d^	IMPORTANT
**Probing Pocket Depth (assessed with: mm)**												
3	non-randomised studies	not serious	not serious	not serious^a^	not serious^b,c,d^	none	173	167	-	MD **0.88 SD higher** (0.82 higher to 0.95 higher)	⨁⨁◯◯ Low^a,b,c,d^	IMPORTANT
**Gingival Index**												
4	non-randomised studies	not serious	not serious	not serious^a^	not serious^b,c,d^	none	241	247	-	MD **0.15 SD higher** (0.11 higher to 0.19 higher)	⨁⨁◯◯ Low^a,b,c,d^	IMPORTANT

*CI* confidence interval, *MD* mean difference

## Discussion

This first part of systematic review and meta-analysis provides an overview of oral health in patients with cleft lip and/or palate. We wanted to determine the effect of cleft lip and/or palate on periodontal health. To do this, we reviewed 24 studies on periodontal parameters and oral microflora, 18 of which underwent meta-analysis. Of note, the studies by Sundel (2018) [19] and Sundel/Nilsson (2016) [26] involved the same sample; therefore, the results of the meta-analysis were considered only once.

Healthy periodontal tissue is essential for successful treatment outcome, especially since each step in cleft treatment can have negative consequences for periodontal health [27–30].

In recent years, oral health monitoring has played a key role in the management of various systemic diseases [31, 32] and maintaining good oral hygiene practices and addressing dental problems promptly can help prevent the exacerbation of existing systemic diseases [33, 34].

This integrated approach to health care emphasizes the interconnectedness of oral and overall health, highlighting the need for collaborative efforts between dental and medical professionals to ensure comprehensive patient care.

Achieving optimal oral hygiene can be difficult due to schisis, soft tissue folds, poorly developed bone, malocclusion, inadequate vestibule depth, and retention area. The potential habitat for bacteria and other microorganisms raises the risk of intraoral translocation of pathogens and periodontal illness. Moreover, patients often undergo multiple surgeries (medical, prosthetic, orthodontic), which pose added risk for periodontal disease [30–38]. Residual scar tissue after defect closure and postsurgical complications (e.g., oronasal fistula) can make oral hygiene difficult to achieve and maintain, leading to poor oronasal health and a higher risk of dental caries, halitosis, and periodontal disease [39–42].

Meta-analysis of the gingival index (GI) and the bleeding index (BI) showed significantly higher values for subjects with cleft lip and/or palate (GI *p* < 0.00001; BI *p* < 0.0001).

One study was included in the qualitative but not the meta-analyses because it applied parameters (bleeding on probing) that cannot be compared with those entered in the statistical analyses. The study suggested that the difference in scores between patients with orofacial cleft and healthy controls was not statistically significant [19]. In a split-mouth study, Plakwicz (2017) observed gingival inflammation more often involving the teeth on the cleft side than on the healthy side, with statistically higher scores for the distovestibular surface of the central incisor (58.8% vs 26.5%), vestibular surface of the canines (38.5% vs 9.7%), and mesiopalatal surface of the canines (50% vs 16.1%) [29]. Patients with cleft lip and/or palate are disposed to a higher risk of gingival inflammation because of the anatomical peculiarity of the cleft area and surgical repair. During the first years of life, the parents of patients with orofacial cleft understandably direct enormous attention to surgical repair, psychological health, and phonetic and phonological development of their children but they tend to neglect teaching them the basics of oral health care, the lack of which can promote inflammation of the superficial periodontium [43, 44].

Furthermore, Plakwicz (2017) found a deeper probing depth for the teeth on the schisis side than on the healthy side, with significantly higher scores (*p* < 0.05) on the vestibular, distopalatal, palatal, and mesiopalatal surfaces of the canines and on the mesiopalatal surfaces of the lateral incisors [29]. The meta-analysis showed higher GI scores for the subjects without labiopalatalschisis (*p* < 0.0000). This observation is shared by Marzouk (2022) [3].

The loss of clinical attachment level (CAL) is greater in patients with cleft lip and palate than in healthy controls (*p* < 0.00001). Mutthineni (2010) found that periodontal status is influenced by schisis type [28]. A split-mouth study (2017) by Plakwicz reported that loss of CAL was greater for teeth on the cleft side than on the healthy side [29]. Mutthineni (2010) compared the sites of teeth adjacent to an alveolar cleft with control sites (not on the cleft side) and found a long supracrestal connective tissue attachment (SCTA) at the sites close to the cleft. Despite the lower bone level, the resistance to periodontal disease seemed to be the same for all teeth [28]. While periodontal disease is usually rare in younger patients, even a minor loss of CAL can signal the initial onset of disease [29].

Evaluation of the development of periodontal disease was reported by prospective studies with long-term follow-up. Salvi (2003) [38] and Huynh-Ba (2009) [39] evaluated the progression of periodontal disease in subjects with orofacial cleft lip and palate not undergoing support periodontal therapy and found an increase in PPD (0.09–0.57 mm) and CAL (1.52–1.85 mm). Periodontal therapy is fundamental for maintaining a low probing depth and preventing loss of CAL after active therapy [40–42]. The two studies did not include a control group, however.

The relationship between cleft lip and palate, periodontal parameters, and the microbiota has been recently revised. The focus of the present study, which differs from that of other reviews, was directed at studies published in the last 10 years, so as to provide an updated overview of the oral health of these patients. We noted that the case–control studies involving patients with orofacial cleft lip and palate and their oral health are few but the revisions on the topic are plentiful. Furthermore, the literature is highly heterogeneous in quality, age group, number of samples, schisis typology, association-exclusion of syndrome history (not always specified), treatment stage, socioeconomic status, geographic area, and types of parameters considered.

### Study limitations

Many studies were unclear about the type, number, and timeframe of the surgical procedures.

The subgroups should take into consideration multiple factors, such as the different types of clefts, associated syndromic pictures, non-syndromic cleft lip and palate, groups of subjects who have/have not undergone surgery, groups of subjects who have not/are having/have already undergone an orthodontic treatment. In addition, few studies mentioned orthodontic treatment, though it may be assumed that only the group with schisis underwent treatment or was planned to. These factors, which are associated with the scarcity of the available literature, are a limitation of the present study.

Most studies do not carry out separate analyses, which does not allow for a precise assessment. For example, both the presence of malocclusion and orthodontic treatment, which plays a key role in the multidisciplinary treatment of subjects affected by clefts, can modify the oral environment the periodontal apparatus, the oral microbiota, the composition and biochemical properties of saliva. A further limitation is that only studies published in English were included and this may have reduced the sample size.

The paper includes studies carried out in several countries, including developing ones, and this represents a strength of this review because it allows us to provide a representative analysis at a global level. However, due to the nature of the studies, the influence of the socio-economic data is not well distinguishable from the other parameters and could have produced an altered result which underestimates the risk of incidence of caries pathology in the poorest areas and overestimates it compared to the richest areas.

Given the paucity of studies in this area, standardized studies using the most recent classification of periodontal illness, with control groups, long-term follow-up, larger sample size, and more homogeneous age groups are warranted.

### Future research directions

To deepen the topic of caries pathology in subjects affected by clefts, standardized case–control studies would be necessary that use the same caries evaluation system, while to deepen the topic of periodontal disease in subjects with clefts, standardized studies using the most recent classification of periodontal disease, with control groups and long-term follow-up, would be necessary. It may be speculated that children with cleft, who are colonized early by periodontium pathogenic species, are at greater risk for periodontal disease. This makes prevention protocols necessary, with regular follow-up visits starting at an early age.

Further research is recommended which includes a greater number of patients, more homogeneous samples, and which takes into consideration more factors, such as types of cleft, orthodontics and surgery. Furthermore, it would be desirable to introduce a standardized method for measuring the microbiota in order to be able to perform a meta-analysis of the microflora in the future.

## Conclusions

The findings of this first part of systemic review and meta-analysis are shared by previous studies that reported that persons with orofacial schisis are at higher risk for poor oral health and dental and periodontal disease. Optimal oral hygiene is often difficult to achieve and maintain. Multiple factors contribute to the risk of dental-periodontal infection. Controversy surrounds the extent to which these risk factors, either singularly or combined, promote co-infection by pathogenic strains and dysbiosis of the oral microbiota, dental caries and periodontal disease.

Given that persons with orofacial cleft lip and palate are at risk of dental-periodontal disease, the implementation is warranted of primary prevention strategies to promote oral health in children with cleft lip and palate starting from a very early age. There is a need for more research with larger and more homogeneous samples, global analyses, and subgroups. It would also be desirable to develop a standardized method to measure oral microbiota and conduct a meta-analysis of the findings of future studies.

## Data Availability

Data are available at the corresponding authors upon reasonable request.
